# Deletions of *LPL* and *NKX3.1* in Prostate Cancer Progression: Game Changers or By-Standers in Tumor Evolution

**DOI:** 10.3390/biom15060758

**Published:** 2025-05-24

**Authors:** Tereza Vodičková, Mária Wozniaková, Vladimír Židlík, Jana Žmolíková, Jana Dvořáčková, Adéla Kondé, Jana Schwarzerová, Michal Grepl, Jan Bouchal

**Affiliations:** 1 Institute of Clinical and Molecular Pathology and Medical Genetics, University Hospital Ostrava and Medical Faculty Ostrava, 703 52 Ostrava, Czech Republic; tereza.vodickova@fno.cz (T.V.); maria.wozniakova@fno.cz (M.W.); jana.schwarzerova2@fno.cz (J.S.); 2 Department of Clinical and Molecular Pathology, Institute of Molecular and Translational Medicine, Palacky University and University Hospital Olomouc, 779 00 Olomouc, Czech Republic; 3 EUC Laboratories CGB, a.s., 703 00 Ostrava, Czech Republic; jana.zmolikova@euclaboratore.cz (J.Ž.); jana.dvorackova@euclaboratore.cz (J.D.); 4 Department of Applied Mathematics, Faculty of Electrical Engineering and Computer Science, VSB—Technical University of Ostrava, 708 00 Ostrava, Czech Republic; adela.konde@vsb.cz; 5 Department of Deputy Director for Science, Research and Education, University Hospital Ostrava, 703 00 Ostrava, Czech Republic; 6 Department of Biomedical Engineering, Faculty of Electrical Engineering and Communication, Brno University of Technology, 616 00 Brno, Czech Republic; 7 Molecular Systems Biology (MOSYS), Department of Functional and Evolutionary Ecology, Faculty of Life Sciences, University of Vienna, 1030 Vienna, Austria; 8 Department of Urology, University Hospital Ostrava, 703 52 Ostrava, Czech Republic; michal.grepl@fno.cz

**Keywords:** prostate cancer, LPL, NKX3.1, immunohistochemistry, FISH, whole-genome sequencing

## Abstract

The tumor suppressor gene *NKX3*.1 and the *LPL* gene are located in close proximity on chromosome 8, and their deletion has been reported in multiple studies. However, the significance of *LPL* loss may be misinterpreted due to its co-deletion with *NKX3.1*, a well-established event in prostate carcinogenesis. This study investigates whether *LPL* deletion represents a biologically relevant event or occurs merely as a bystander to *NKX3.1* loss. We analyzed 28 formalin-fixed paraffin-embedded prostate cancer samples with confirmed *LPL* deletion and 28 without. Immunohistochemical staining was performed, and previously published whole-genome sequencing data from 103 prostate cancer patients were reanalyzed. Deletion of the 8p21.3 region was associated with higher Gleason grade groups. While NKX3.1 expression was significantly reduced in prostate cancer compared to benign prostatic hyperplasia, LPL protein expression showed no significant difference between cancerous and benign tissue, nor was it affected by the 8p21.3 deletion status. Copy number analysis confirmed the co-deletion of *NKX3.1* and *LPL* in 54 patients. Notably, *NKX3.1* loss without accompanying *LPL* deletion was observed in eight additional cases. These findings suggest that *LPL* deletion is a passenger event secondary to *NKX3.1* loss and underscore the importance of cautious interpretation of cytogenetic findings involving the *LPL* locus.

## 1. Introduction

Although the incidence of prostate cancer continues to rise and its mortality rate remains steady, it is now widely recognized as a manageable disease. The ability to diagnose prostate cancer at an early stage has significantly contributed to this trend, allowing for effective treatment before metastasis occurs. However, prostate cancer remains a major health burden and is the second-leading cause of cancer death in developed countries [1,2].

The *NKX3.1* gene is a tumor suppressor gene that plays a key role in regulating prostate development and function, helping to prevent tumor formation. The *NKX3.1* gene regulates prostate cell proliferation, which is important for maintaining regular prostate size [3]. It also supports the differentiation of prostate cells, which is essential for normal prostate functioning. It is involved in DNA damage repair, a critical process for preventing mutations that could lead to tumor formation. NKX3.1 interacts with other proteins involved in cellular processes such as the cell cycle and apoptosis [4]. Mutations or reduced expression of NKX3.1 can dysregulate these processes and may contribute to the development of prostate cancer. Therefore, NKX3.1 is considered a key factor in maintaining healthy prostate and preventing its malignant transformation [5]. Deletion of the *NKX3.1* gene disrupts normal prostate epithelium, initiating the oncogenic cascade [3].

The *LPL* gene encodes the enzyme lipoprotein lipase, which is crucial for lipid metabolism. This enzyme hydrolyzes triglycerides in lipoproteins (such as chylomicrons and very low-density lipoproteins—VLDL), releasing fatty acids and glycerol to be used by the body’s cells. LPL activity is essential for maintaining normal levels of triglycerides and other lipids in the blood [6]. Mutations in the *LPL* gene can lead to hyperlipidemia, which increases the risk of atherosclerosis and cardiovascular diseases. The released fatty acids are either oxidized for energy or stored in adipose tissue, making lipoprotein lipase a significant player in energy metabolism and fat storage [7]. Lipoprotein lipase in the prostate contributes to local lipid metabolism [8]. It hydrolyzes triglycerides into free fatty acids and glycerol, which can be used for energy or as building blocks for cell membrane synthesis. Fatty acids obtained through LPL activity can serve as signaling molecules that regulate cell proliferation and growth, which is important for maintaining the health and regeneration of prostate tissue [9]. In the context of prostate cancer, increased LPL activity may promote tumor cell growth. Tumor cells often display altered lipid metabolism, characterized by increased lipolysis and the use of fatty acids as both an energy source and building blocks for rapid cell division. Lipids and fatty acids can also influence hormonal signaling in the prostate, which is sensitive to androgens. In this way, LPL can indirectly affect the growth and function of prostate cells by modifying the lipid environment [10].

*NKX3.1* is located in the 8p21.2 region, while *LPL* is in the 8p21.3 region, only 3.7 Mbp apart [11]. An established cytogenetic probe against the *LPL* locus has been repeatedly used in prostate cancer research [9,12,13,14]. This study aims to demonstrate that deletion of the *LPL* gene, frequently mentioned in prostate cancer research, occurs incidentally alongside the tumor suppressor *NKX3.1*, a well-known event in prostate carcinogenesis. This finding could enhance the understanding and interpretation of cytogenetic results with the *LPL* probe.

## 2. Materials and Methods

### 2.1. Patients

Our cohort includes 56 patients (Table 1), who were assessed for the expression of NKX3.1 and LPL proteins by immunohistochemistry on the same paraffin blocks used for FISH testing (Table S1). Twenty-eight patients with a confirmed *LPL* (8p21.3) deletion were selected, along with 28 control patients with normal status.

The second cohort has previously been published by Camacho et al. [15]. These authors performed the whole-genome DNA sequencing of 103 prostate cancer patients, and the most frequent copy number variation (loss in 62 patients) was found at 8p21.3–p21.2, where 16 genes are located, including *NKX3.1* and *LPL* (Table S2). The corresponding author, Professor Daniel S. Brewer, kindly provided us with the complete data, which enabled us to perform our reanalysis (see below).

### 2.2. Immunohistochemistry

Immunohistochemistry was conducted on formalin-fixed, paraffin-embedded (FFPE) samples obtained from radical prostatectomy. Paraffin sections, 5 μm thick and stretched on electrostatic slides, underwent staining using a Ventana BenchMark Ultra automated stainer (Roche, Rotkreuz, Switzerland). The monoclonal rabbit antibody NKX3.1 (clone EP356, catalogue number 07859759001, ready-to-use, Cell Marque, Roche, Rotkreuz, Switzerland) was applied with an incubation period of 20 min. Additionally, the monoclonal mouse LPL antibody (clone OTI3A10, catalogue number NBP2-01395, Novus Biological, Centennial, CO, USA) was diluted at 1:150, with an incubation time of 60 min. The evaluation of both proteins utilized the final histoscore, derived from the product of the percentage of positive cells and their staining intensity. Positive cells were categorized into intervals: 0 (negative), 25 (1–25% positive cells), 50 (26–50% positive cells), 75 (51–75% positive cells), and 100 (76–100% positive cells). Staining intensity was scored on a scale of 0 (negative), 1 (weakly positive), 2 (moderately positive), and 3 (strongly positive). Expression assessment was performed in the prostate cancer and benign prostatic hyperplasia. Using appropriate statistical methods, we also examined the correlation between age and Gleason Grade Groups for both NKX3.1 and LPL.

### 2.3. FISH

Fluorescence in situ hybridization (FISH) was performed on the same blocks used for immunohistochemistry. Prior to hybridization, slides are pretreated following Kreatech’s tissue Digestion Kit (Leica Biosystems, Deer Park, TX, USA) protocol: Bake the slides at 80 °C for 1–2 h. Deparaffinize the warm slides by soaking in xylene for 2 × 8 min. Rehydrate the slides by soaking them in 100%, 85%, and 70% ethanol for 3 min each. Wash with dH2O for 3 min at room temperature. Place slides in 0.01 M sodium citrate at 96–98 °C for 15 min. Rinse in dH2O for 3 min at room temperature. Cover the paraffin section with Pepsin Solution and incubate at room temperature for 30–35 min. Dehydrate the slides by soaking in 70%, 80%, and 100% ethanol for 1 min each. Air-dry and apply 10 µL of LPL/MYC/SE 8 CP probe (catalogue number KBI-00114, Kreatech Diagnostics, Leica Biosystems, Deer Park, TX, USA) on the paraffin section and cover with a cover slip. The coverslip was sealed with Fixogum rubber cement. Slide and probe were co-denatured at 80 °C for 5 min, followed by overnight hybridization at 37 °C in the ThermoBrite. The next morning, the slides were washed for 2 min in 0.4× SSC, 0.3% NP-40 at 72 °C for 2 min, and subsequently for 2 min in 2× SSC, 0.1% NP-40 at room temperature. Finally, slides were dehydrated in 70%, 80%, and 100% ethanol, air-dried and embedded using DAPI/Antifade, and covered with cover glass. The evaluation was based on 50–100 tumor nuclei and included the following criteria: Normal finding: <10% of cells with 3 or more signals and <55% of cells with 1 or 0 signals for CEP 8. Loss 8: >55% of cells with 1 or 0 signals for CEP 8. Loss 8p22: 8p22/CEP8 signal ratio < 0.85.

### 2.4. Reanalysis of WGS Data

We reanalyzed the whole-genome sequencing (WGS) data originally published by Camacho et al. [15], focusing on copy number variation (CNV) profiles. From their dataset, we selected patients who showed deletions on chromosome 8 that overlapped both of our genes of interest. The CNV regions were identified using their publicly available CNV call files, which we filtered based on genomic coordinates corresponding to these loci.

For downstream analysis, we used R (version 4.3.2) and several specialized packages. We used R\CNViz (version 1.14.0) [16] to visualize and interpret CNV calls, including plotting genome-wide and locus-specific copy number changes. The R\ggplot2 package (version 3.5.1) [17] was used for generating custom plots to illustrate CNV patterns across patient samples. Additionally, R\gggenes (version 0.5.1) was employed to create gene structure diagrams to map the positions of the genes of interest within the deleted regions.

### 2.5. Statistical Analysis

Numerical variables are presented as medians and ranges. Categorical variables are introduced as absolute and relative frequencies (%). Between-group differences are analyzed using the Mann–Whitney test or Fisher’s exact test. The paired Wilcoxon test is used to analyze differences in NKX3.1 and LPL protein histoscore between benign prostatic hyperplasia and prostate cancer. The association of selected parameters was visualized using a 100% stacked bar plot or paired boxplots. The significance level was set to 0.05, and the statistical analysis was performed using R software (version 4.3.2) with the maximum available data.

## 3. Results

### 3.1. LPL (8p21.3) Deletion Is Associated with a Poor Gleason Score

We analyzed a cohort of prostate cancer patients examined by FISH between 2007 and 2017, consisting of 28 patients with confirmed *LPL* (8p21.3) deletion and 28 patients without this aberration. Clinicopathological data are summarized in Table 1.

Notably, Gleason Grade Groups were significantly associated with the deletion status (*p* = 0.009, Table 1). Among patients without the *LPL* (8p21.3) deletion, less aggressive GG1–GG2 were predominant (86% of cases), while GG3–GG5 were found only in 14% of cases. However, among patients with the *LPL* (8p21.3) deletion, the percentage of GG3–GG5 (Gleason score 4 + 3 and higher) increased to 50%. This information suggests an association between the *LPL* (8p21.3) deletion and worse Gleason score, i.e., 4 + 3 and higher (GG3-GG5; Table 1).

### 3.2. Expression of NKX3.1 Is Decreased in Cancer in Comparison to Benign Prostatic Hyperplasia, but Without Relation to 8p21.3 Deletion

**Figure 1:**
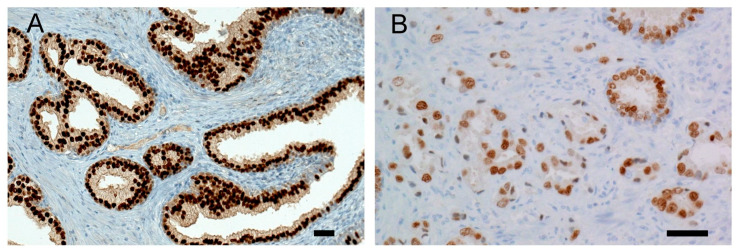
Representative NKX3.1 immunohistochemistry: (**A**) Strong nuclear positivity of NKX3.1 is present in benign prostatic hyperplasia, histoscore 300. (**B**) Prostate cancer cells show reduced expression of NKX3.1 protein, histoscore 100. Malignant glands are smaller, irregular, more crowded, and lack branching and papillary infoldings. A scale bar represents 50 μm.

**Figure 2:**
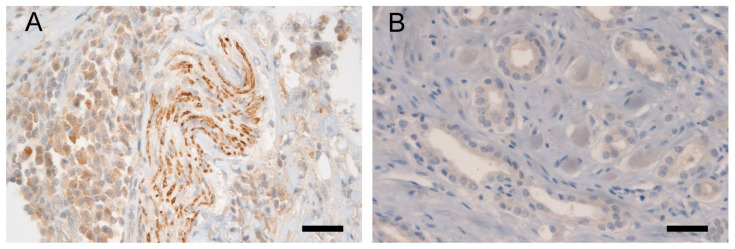
Representative LPL immunohistochemistry: (**A**) Validation of the anti-LPL antibody in bladder tissue, showing positivity in nerve bundles (in the middle of the picture) and in bladder cells (predominantly on the left side in the picture); (**B**) Weak cytoplasmic expression of LPL protein in malignant cells of irregular glands in adenocarcinoma of the prostate, histoscore 50. A scale bar represents 50 μm.

**Figure 3:**
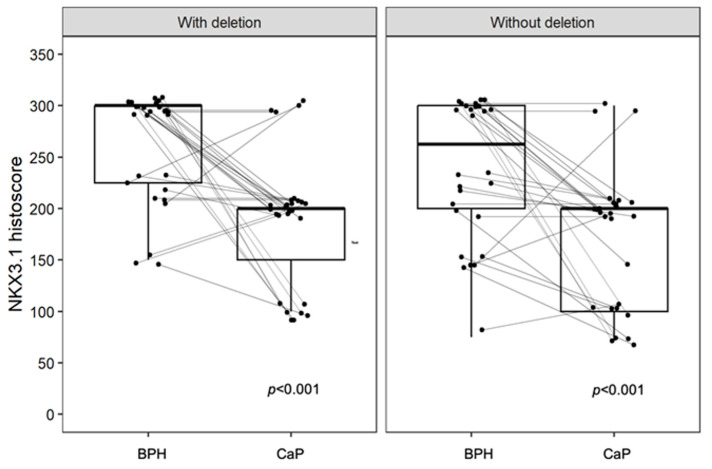
The expression of NKX3.1 is decreased in CaP but shows no significant association with deletion status. A significant difference is observed in BPH compared to CaP in both groups, with a *p*-values < 0.001. Without deletion, there is a larger overlap in the distribution of data within the box plot, whereas with deletion, the data distribution is more distinctly separated. Box plots represent the median, 25–75% percentiles, and range of values. Histoscores for individual patients with BPH and CaP are also shown.

**Figure 4:**
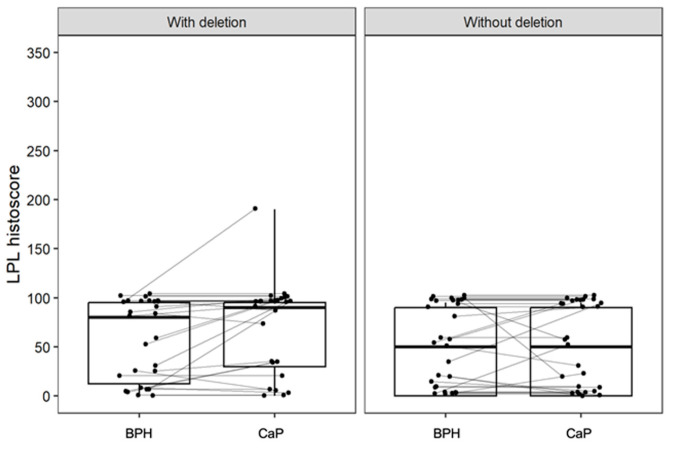
The expression of LPL is low and is not significantly different between groups with and without deletion, and with respect to the tissue architecture (BPH or CaP). Box plots represent the median, 25–75% percentiles, and range of values. The histoscores for individual patients with BPH and CaP are also shown.

Immunohistochemistry for NKX3.1 and LPL proteins was performed on the same tissue blocks used for FISH (Figure 1 and Figure 2). NKX3.1 histoscore was significantly lower in carcinoma compared to benign hyperplasia in both patient groups, with (*p* < 0.001) and without 8p21.3 deletion (*p* < 0.001) (Figure 3). In contrast, LPL expression was very low and showed no significant association with tissue structure, cancer presence, or deletion status (Figure 4).

**Figure 5:**
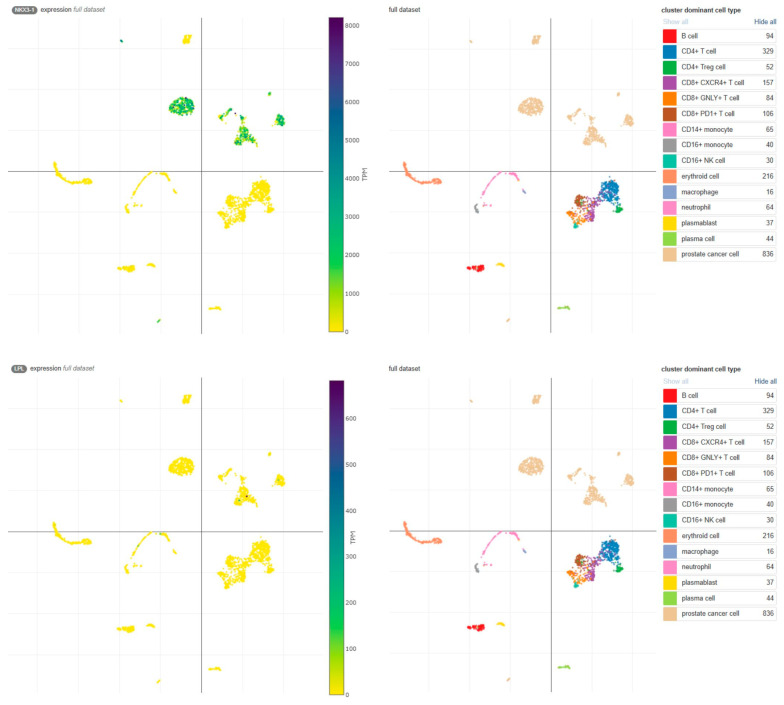
Expression of NKX3.1 and LPL transcripts in the Single Cell Portal [18]. High NKX3.1 expression is observed in prostate cancer cells (**upper panel**), whereas LPL expression is limited to a few cells (**lower panel**). Data were obtained from the study ‘Transcriptional mediators of treatment resistance in lethal prostate cancer’ [19]. TPM, transcripts per million.

Similarly, low LPL expression was observed in publicly available single-cell sequencing data (Figure 5, [18,19]). These findings highlight the significant reduction in NKX3.1 protein expression, but not LPL, during the transition from benign to malignant prostate tissue, potentially driven by deletion and other mechanisms.

### 3.3. Whole-Genome Copy Number Analysis Shows Co-Deletion of LPL and NKX3.1 Genes

**Figure 6:**
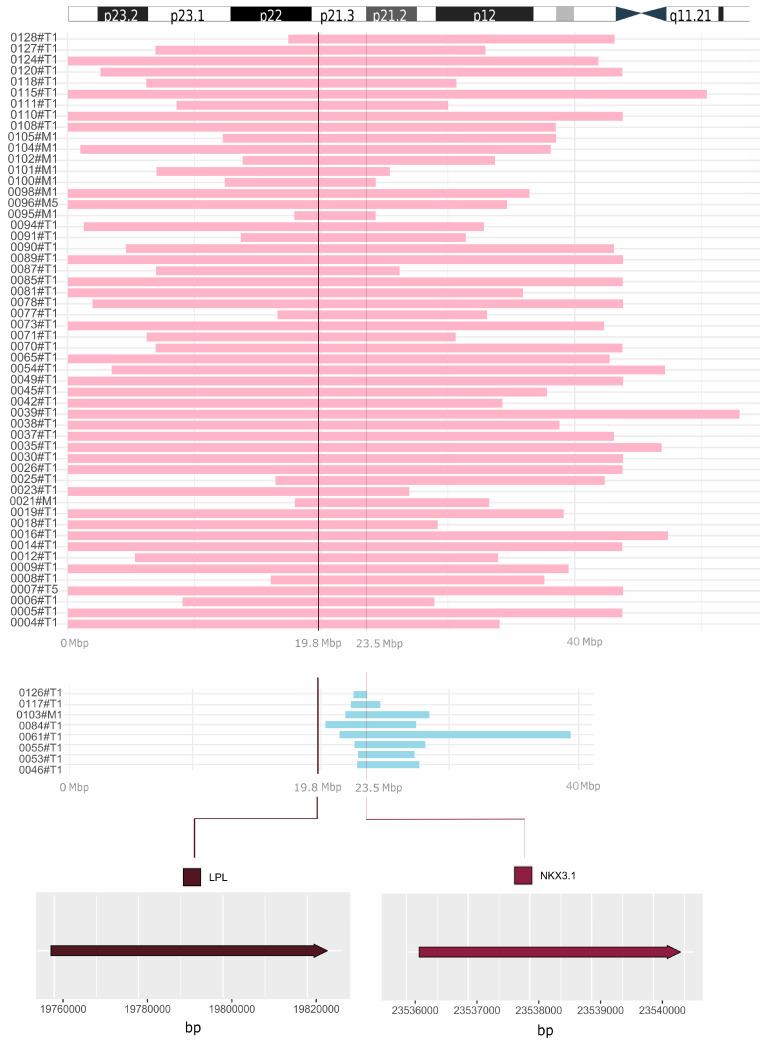
Comprehensive visualization of deletions unveiled by reanalysis of WGS data. Co-deletion of *NKX3.1* and *LPL* is displayed in pink (54 prostate cancer patients). The remaining 8 patients lost the *NKX3.1* gene, but not *LPL* (blue reads). In the upper part of the figure, the short arm of chromosome 8 is depicted for a closer orientation of the position of the *LPL* and *NKX3.1* genes. At the bottom, the exact locations of the *LPL* (chr8: 19,796,764–19,824,770) and *NKX3.1* (chr8: 23,536,206–23,540,451) genes are shown. On the left, designation of samples from the study by Camacho et al. is provided [15]. T, tumor; M, metastasis; Mbp, megabase pairs.

We did not perform a genetic analysis on our cohort. However, detailed results were publicly available from the whole-genome DNA sequencing of 103 prostate cancer patients [15]. The most frequent copy number variation (loss in 62 patients) was found at 8p21.3–p21.2, where 16 genes are located, including *NKX3.1.* Supplementary Materials for chromosome 8 also clearly displayed large deletions, affecting many more genes, presumably also the *LPL* [15]. Our reanalysis of complete data confirmed the co-deletion of *NKX3.1* and *LPL* in 54 patients (Figure 6, pink reads). The remaining eight patients lost the *NKX3.1* gene, but not *LPL* (Figure 6, blue reads).

## 4. Discussion

Our study presents a statistically significant association between the 8p21.3 deletion and the tumor’s Gleason Grade Groups. Among patients with the deletion, Gleason Grade Groups GG1–GG2 were as prevalent as GG3–GG5, whereas in patients without the deletion, GG1–GG2 predominated, with GG3–GG5 being less common. Similarly, Trock et al. addressed this relationship in their 2016 study, focusing on the GG3 component and various Gleason scores [20]. Gallucci et al. also reported a statistically significant relationship between the deletion of 8p21 and higher Gleason score [8]. Importantly, Kluth et al. performed a large study on chromosome 8 deletions with respect to prostate cancer prognosis [21]. Typically, these deletions involved the entire short arm of chromosome 8 and were associated with both an adverse Gleason score and shorter biochemical recurrence. The prognostic value of 8p deletion was further enhanced when *PTEN* deletion was also present (10q23.31).

Additionally, our study demonstrates a decline in the NKX3.1 histoscore in cancer compared to benign hyperplasia, irrespective of the presence or absence of the 8p21.3 deletion. Bethel et al. reported that NKX3.1 expression is reduced in atrophic areas, prostatic intraepithelial neoplasia (PIN), and adenocarcinoma compared to normal epithelium [22]. Similarly, our previous study also observed lower NKX3.1 intensity in PIN and prostate cancer regions in comparison to BPH [23]. While Bethel et al. identified an association between the 8p21.3 deletion and reduced NKX3.1 expression [22], our study did not reproduce this finding, potentially due to variations in immunohistochemical staining resulting from the slow fixation of prostate tissues [23,24]. Moreover, NKX3.1 expression may be influenced by methylation [25]. A substantial fraction of NKX3.1 target genes also overlap with direct targets of the oncoprotein Myc. It has been shown that NKX3.1 depletion cooperates with Myc overexpression to promote prostate cancer in transgenic mice [26]. Conversely, a certain level of NKX3.1 expression is retained even in metastatic lesions [27] and is essential for the survival of androgen-dependent prostate cancer cells [28]. Overall, NKX3.1 appears to function as a haploinsufficient tumor suppressor gene, where its reduced expression compromises normal cellular functions, contributing to tumor development [29,30].

Expression of LPL was low and without significant association with the tissue structure or the deletion status. Considering the importance of lipid metabolism for steroidogenesis and cancer growth [31], enhanced expression of LPL has been reported in advanced prostate cancer cell lines [32]. On the other hand, Kim et al. explored the hypothesis that LPL may be a tumor suppressor gene, inactivated by somatic deletion and hypermethylation in prostate cancer [33]. Hemizygous deletion of the *LPL* gene was observed in 45% of tumor samples, with promoter hypermethylation occurring in 45% of cases with the deletion and 22% without it. Kuemmerle et al. observed perinuclear LPL positivity in the small immunohistochemistry analysis of ten samples with their in-house antibody [34]. Importantly, they also found low LPL expression in all prostate cancer cell lines, which they explained with the frequent loss of the *LPL* locus due to a nearby tumor suppressor gene, however, without naming *NKX3.1* [35].

Camacho et al. reported loss of the 8p21.3–8p21.2 region, which encompasses *NKX3.1* and *LPL*, as the most frequent deletion in 62 out of 103 prostate cancer patients [15]. By reanalyzing the complete data, we showed the co-deletion of *NKX3.1* and *LPL* in 54 out of 62 patients. Importantly, the remaining eight patients lost the *NKX3.1* gene, but not *LPL*. The findings support the well-known role of NKX3.1 as a prostate tumor suppressor, while the *LPL* gene is often deleted alongside *NKX3.1* due to their close proximity. Thus, the designation of *LPL* for the 8p21.3 probe may be considered misleading.

## 5. Conclusions

NKX3.1 expression was significantly lower in carcinoma than in benign hyperplasia, highlighting its role in cancer progression. In contrast, LPL expression was very low and showed no difference between BPH and cancer. While 8p21.3 deletion was associated with a poor Gleason score, it was not linked to NKX3.1 or LPL expression in our prostate cancer cohort. Beyond genetic loss, other mechanisms may contribute to NKX3.1 downregulation during carcinogenesis. Notably, genome copy number analysis confirmed the co-deletion of *NKX3.1* and *LPL* in 54 of 62 patients, while the remaining 8 patients lost *NKX3.1* but retained *LPL*. These findings reinforce NKX3.1’s well-established role as a prostate tumor suppressor, while suggesting that *LPL* is frequently deleted due to its proximity rather than functional significance. Thus, designating *LPL* for the 8p21.3 probe may be misleading.

## Figures and Tables

**Table 1:** Patients’ characteristics with known *LPL* (8p21.3) deletion status (N = 56).

	Total (n = 56)	With Deletion (n = 28)	Without (n = 28)	*p*
Age, years, median (range)	63 (52; 73)	62 (56; 72)	65 (52; 73)	0.297
PSA, ng/mL, median (range)	7.5 (2.4; 23.0)	8.1 (2.6; 21.0)	7.0 (2.4; 23.0)	0.491
Gleason Grade Groups, n (%)				0.009
GG1–GG2	38 (68)	14 (50)	24 (86)	
GG3–GG5	18 (32)	14 (50)	4 (14)	
pT, n (%)				0.688
2	36 (64)	16 (57)	20 (71)	
3	18 (32)	11 (39)	7 (25)	
4	2 (4)	1 (4)	1 (4)	
Lymph node metastasis, n (%)				>0.999
0	53 (98)	26 (100)	27 (96)	
1	1 (2)	0 (0)	1 (4)	
Not available	2	2	0	

The values represent the median and the range, or absolute and relative frequencies (%). The *p* -value was obtained with the Mann–Whitney test or Fisher’s exact test.

## Data Availability

The original contributions presented in this study are included in the article. Further inquiries can be directed to the corresponding authors.

## Supplementary Materials

The following supporting information can be downloaded at: https://www.mdpi.com/article/10.3390/biom15060758/s1, Table S1: Results of immunohistochemistry; Table S2: Results of WGS reanalysis.

## Abbreviations

The following abbreviations are used in this manuscript: BPH, benign prostatic hyperplasia; CaP, prostate cancer; FISH, fluorescence in situ hybridization; FFPE, formalin-fixed paraffin-embedded; PIN, prostatic intraepithelial neoplasia; PSA, prostate-specific antigen; pT, pathological staging of the primary tumor; WGS, whole-genome sequencing.
